# Overproduction of the membrane-bound [NiFe]-hydrogenase in *Thermococcus kodakarensis* and its effect on hydrogen production

**DOI:** 10.3389/fmicb.2015.00847

**Published:** 2015-08-26

**Authors:** Tamotsu Kanai, Jan-Robert Simons, Ryohei Tsukamoto, Akihito Nakajima, Yoshiyuki Omori, Ryoji Matsuoka, Haruki Beppu, Tadayuki Imanaka, Haruyuki Atomi

**Affiliations:** ^1^Laboratory of Biochemical Engineering, Department of Synthetic Chemistry and Biological Chemistry, Graduate School of Engineering, Kyoto UniversityKyoto, Japan; ^2^Japan Science and Technology Agency, Core Research of Evolutional Science and TechnologyTokyo, Japan; ^3^Taiyo Nippon Sanso CorporationTokyo, Japan; ^4^Research Organization of Science and Technology, Ritsumeikan UniversityKusatsu, Japan

**Keywords:** hydrogen, hydrogenase, hyperthermophile, archaea, genetic engineering, dark fermentation, *Thermococcus*

## Abstract

The hyperthermophilic archaeon *Thermococcus kodakarensis* can utilize sugars or pyruvate for growth. In the absence of elemental sulfur, the electrons *via* oxidation of these substrates are accepted by protons, generating molecular hydrogen (H_2_). The hydrogenase responsible for this reaction is a membrane-bound [NiFe]-hydrogenase (Mbh). In this study, we have examined several possibilities to increase the protein levels of Mbh in *T. kodakarensis* by genetic engineering. Highest levels of intracellular Mbh levels were achieved when the promoter of the entire *mbh* operon (TK2080-TK2093) was exchanged to a strong constitutive promoter from the glutamate dehydrogenase gene (TK1431) (strain MHG1). When MHG1 was cultivated under continuous culture conditions using pyruvate-based medium, a nearly 25% higher specific hydrogen production rate (SHPR) of 35.3 mmol H_2_ g-dcw^−1^ h^−1^ was observed at a dilution rate of 0.31 h^−1^. We also combined *mbh* overexpression using an even stronger constitutive promoter from the cell surface glycoprotein gene (TK0895) with disruption of the genes encoding the cytosolic hydrogenase (Hyh) and an alanine aminotransferase (AlaAT), both of which are involved in hydrogen consumption (strain MAH1). At a dilution rate of 0.30 h^−1^, the SHPR was 36.2 mmol H_2_ g-dcw^−1^ h^−1^, corresponding to a 28% increase compared to that of the host *T. kodakarensis* strain. Increasing the dilution rate to 0.83 h^−1^ or 1.07 h^−1^ resulted in a SHPR of 120 mmol H_2_ g-dcw^−1^ h^−1^, which is one of the highest production rates observed in microbial fermentation.

## Introduction

In view of the high demand for renewable energy resources, biological hydrogen (H_2_) produced by photosynthetic and anaerobic fermentative microorganisms is a promising biofuel that has attracted research activities during the last decades (Hallenbeck, 2009; Oh et al., 2011; Rittmann et al., 2015). Light-dependent H_2_ production processes by photosynthetic organisms have been limited by their low cell-specific productivities, and by the requirement of large reactor surface areas for light exposure (Melis et al., 2000; Akkerman et al., 2002; Lo et al., 2010). In contrast, dark fermentation by fermentative anaerobes revealed higher productivities, and studies mostly focused on anaerobic cultures of mesophilic bacteria such as *Enterobacter* and *Clostridium* (Taguchi et al., 1995; Kumar and Das, 2001; Rittmann and Herwig, 2012), (hyper-) thermophilic bacteria such as *Thermotoga* and *Caldicellulosiruptor* (van Niel et al., 2002; Mars et al., 2010) and hyperthermophilic archaea, especially of the order Thermococcales, such as *Pyrococcus* and *Thermococcus* (Schicho et al., 1993; Kanai et al., 2013; Bae et al., 2015).

The hyperthermophilic archaeon *T. kodakarensis* grows on media with pyruvate or carbohydrates (such as soluble starch or maltodextrin) (Morikawa et al., 1994; Atomi et al., 2004). It displays one of the highest cell-specific H_2_ production rates when grown in a continuous culture (up to 60 mmol g-dcw^−1^ h^−1^) with pyruvate (Kanai et al., 2005). Using similar continuous culture conditions, even higher H_2_ production rates were reported for *Pyrococcus furiosus* (up to 102 mmol g-dcw^−1^ h^−1^ with maltose) (Schicho et al., 1993). Recently, a maximum cell-specific H_2_ production rate of 352 mmol g-dcw^−1^ h^−1^ with formate was reported in a batch culture of *Thermococcus onnurineus* (Bae et al., 2015). Bacteria typically exhibit maximum cell-specific H_2_ production rates below 40 mmol g-dcw^−1^ h^−1^ (Rittmann and Herwig, 2012), but have the advantage to reach higher cell densities.

**Figure 1:**
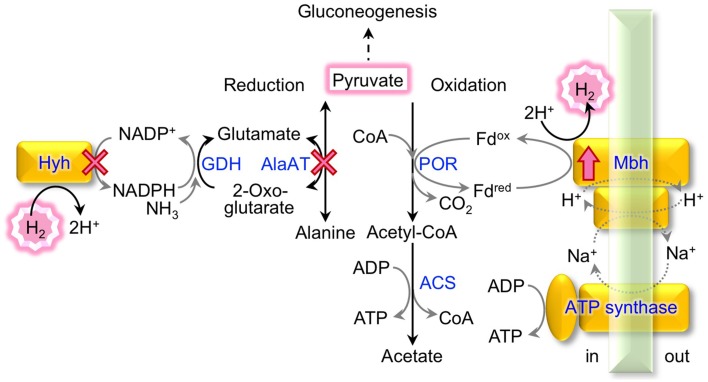
**Pyruvate conversion and H_2_ metabolism in *T. kodakarensis***. The metabolic pathways of pyruvate reduction to alanine linked to H_2_ consumption and of pyruvate oxidation to acetate linked to H_2_ production are indicated. Enzymes marked with a cross were deleted and the Mbh was overproduced in this study; ACS, acetyl-CoA synthetase; AlaAT, alanine aminotransferase; GDH, glutamate dehydrogenase; Hyh, cytosolic [NiFe]-hydrogenase; Mbh, membrane-bound [NiFe]-hydrogenase; POR, pyruvate:ferredoxin oxidoreductase.

In *T. kodakarensis*, cultivation on pyruvate was shown to promote a 44% higher cell specific H_2_ production rate than cultivation on soluble starch (Kanai et al., 2005). Many enzymes involved in pyruvate metabolism and H_2_ production of Thermococcales were identified in *P. furiosus* (Verhees et al., 2003; Bräsen et al., 2014) and genome analysis of *T. kodakarensis* confirmed the presence of equivalent pathways in this organism (Fukui et al., 2005). Besides being used as starting material for gluconeogenesis, pyruvate is mainly either reduced to alanine *via* alanine aminotransferase (AlaAT) (Ward et al., 2000), or is oxidized to acetate (Figure 1).

**Figure 2:**
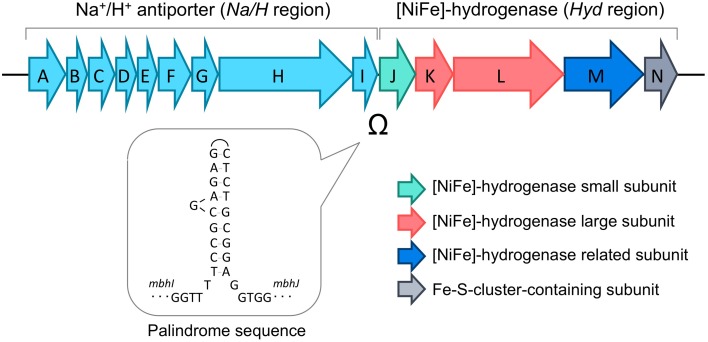
**Gene structure of the membrane-bound [NiFe]-hydrogenase complex (Mbh)**. The palindromic sequence found between the genes encoding the Na^+^/H^+^ antiporter (*Na/H* region, *mbhA-I*) and the genes encoding the catalytic hydrogenase (*Hyd* region, *mbhJ-N*) are indicated.

Pyruvate oxidation comprises two steps catalyzed by pyruvate:ferredoxin oxidoreductase (POR) (Blamey and Adams, 1993) and acetyl-CoA synthetases (ACSs), which produce ATP through substrate-level phosphorylation (Mai and Adams, 1996; Glasemacher et al., 1997). The POR reaction produces acetyl-CoA and CO_2_, and an electron from this reaction is transferred to oxidized ferredoxin (Fd^ox^) to produce reduced ferredoxin (Fd^red^). A membrane-bound [NiFe]-hydrogenase complex (Mbh; TK2080-TK2093) (Figure 2) utilizes the electrons to produce molecular H_2_ with protons and regenerates Fd^ox^ (Sapra et al., 2000; Silva et al., 2000; Kanai et al., 2011). The metabolism indicates a H_2_/CO_2_ gas production ratio of 1 from pyruvate. The Mbh reaction also contributes to energy conservation as it is thought to be coupled to proton export, which *via* an Na^+^/H^+^-antiporter domain, results in a sodium gradient that fuels ATP synthesis by the A_1_A_0_-ATP synthase (Sapra et al., 2003; Pisa et al., 2007). Deletion of Mbh abolishes H_2_production and impairs growth under H_2_-producing conditions, reflecting that Mbh is the key [NiFe]-hydrogenase that is responsible for H_2_ production in *T. kodakarensis* (Kanai et al., 2011; Santangelo et al., 2011) as well as in *P. furiosus* (Schut et al., 2012).

Pyruvate reduction into alanine potentially competes with H_2_ production from pyruvate. Glutamate, which is used as an amino donor for pyruvate reduction through AlaAT, is regenerated from 2-oxoglutarate *via* glutamate dehydrogenase (GDH) coupled with NADPH consumption (Consalvi et al., 1991; Robb et al., 1992; Yokooji et al., 2013). NADPH is partially regenerated by a cytosolic [NiFe]-hydrogenase complex (Hyh; TK2069-2072), which utilizes H_2_ as an electron donor for NADP^+^ reduction (Bryant and Adams, 1989; Ma et al., 2000; Kanai et al., 2003, 2011). In a continuous, gas exchange culture of *T. kodakarensis* with pyruvate as a substrate, the deletion of *hyh* increases the gas production ratio of H_2_/CO_2_ by 8% (Kanai et al., 2011). An increase in cell-specific H_2_ production of up to three-fold was also reported in a closed batch culture with the same substrates (Santangelo et al., 2011).

Attempts to increase microbial H_2_ production *via* genetic engineering revealed two main successful strategies; overexpression of enzymes directly involved in H_2_ production and the deletion of competing pathways (Yoshida et al., 2005, 2007; Kim et al., 2009; Klein et al., 2010). The effect of homologous overexpression of the H_2_-evolving hydrogenase on cell-specific H_2_ production rates depends on the organism and ranges from no effect (*Clostridium acetobutylicum*) to a 2.8-fold increase (*Escherichia coli*) (Yoshida et al., 2005; Klein et al., 2010). Heterologous overexpression of the membrane-bound formate hydrogen lyase complex of *T. onnurineus* in *P. furiosus* enabled conversion of formate into H_2_ in addition to its native H_2_ production from maltose (Lipscomb et al., 2014). In *E. coli*, overexpression of the hydrogenase from *Enterobacter cloacae* led to H_2_ production levels comparable to those observed in *Enterobacter* species (Chittibabu et al., 2006). The effects of deleting competing pathways (H_2_-consuming hydrogenases, AlaAT) (Kanai et al., 2011; Santangelo et al., 2011) or pathways generating compounds that inhibit H_2_ production have been examined (Kim et al., 2009). For example, the disruption of lactate and succinate generating pathways in *E. coli*, which have a negative effect on H_2_ production, resulted in an increase in cell-specific H_2_ production rates by 1.3-fold (Yoshida et al., 2007).

In the present study, we performed homologous overexpression of the Mbh gene in *T. kodakarensis via* different genetic approaches in combination with the disruption of the genes encoding H_2_-consuming Hyh and AlaAT (Figure 1). The effects on both cell-specific H_2_ production rate (SHPR; mmol H_2_ g-dcw^−1^ h^−1^) and media-volume specific H_2_ evolution rate (HER; mmol H_2_ L^−1^ h^−1^) were analyzed during cultivation with pyruvate under continuous culture conditions.

## Materials and methods

### Microorganisms and culture conditions

*E. coli* DH5α was used for general DNA manipulation and sequencing. *E. coli* strains were cultivated in LB medium (10 g L^−1^ tryptone, 5 g L^−1^ yeast extract and 10 g L^−1^ NaCl) at 37°C. Ampicillin was added to the medium at a concentration of 100 μg mL^−1^.

*T. kodakarensis* strains and plasmids used in this study are listed in Table 1. *T. kodakarensis* strains were routinely grown under anaerobic conditions at 85°C in MA-YT medium with the following composition; 30.4 g L^−1^ Marine Art SF-1 salt as artificial sea salts (Tomita Pharmaceutical, Tokushima, Japan), 5 g L^−1^ yeast extract and 5 g L^−1^ tryptone. In the case of cultivation with S^0^, sulfur powder was added at a concentration of 2 g L^−1^ after autoclaving the MA-YT medium. In the case of cultivation with pyruvate, 5 g L^−1^ sodium pyruvate was added to the MA-YT medium before autoclaving (MA-YT-Pyr).

**Table 1:** **Strains and plasmids used in this study**.

	**Strain or plasmid**	**Relevant characteristics**	**Sources or references**
Strains	KU216	KOD1 Δ*pyrF*	Sato et al., 2005
	MHG1	KU216 *mbh*::P*_*mbh*_*−2μ-P*_*gdh*_*-*mbhA*	This study
	MHC1	KU216 Δ*chiA*::P*_*csg*_*-*mbhJKLMN*-2μ	This study
	MPD1	KU216 *mbh*::ΔΩ	This study
	DPHA1	KU216 Δ*hyhBGSL*::2μ′Δ*aat*::2μ′	Kanai et al., 2011
	MAH1	DPHA1 *mbh*::P*_*mbh*_*-P*_*csg*_*-*mbhA*	This study
Plasmids	pUC118	Amp^*r*^ general cloning vector	Takara Bio (Otsu, Japan)
	pUD	pUC118 derivative; *pyrF* marker cassette	Sato et al., 2003
	pUD2	pUC118 derivative; *pyrF* marker cassette	Sato et al., 2005
	pUP1	pUC118 derivative; 2μ −*pyrF*−2μ	This study
	pMHG1	pUC118 derivative; P*_*mbh*_*−2μ-*pyrF*−2μ- P*_*gdh*_-mbhA*	This study
	pMHC1	pUC118 derivative; *chiAN*-P*_*csg*_*-*mbhJKLMN*−2μ-*pyrF*−2μ-*chiAC*	This study
	pMPD1	pUD2 derivative; ΔΩ	This study
	pMAH1	pUD2 derivative; P*_*mbh*_*-P*_*csg*_*-*mbhA*	This study

### Construction of *T. kodakarensis* mutant strains

Disruption of specific genes by double-crossover homologous recombination (for MHG1 and MHC1) or single-crossover homologous recombination followed by pop-out deletion of region containing *pyrF* marker (for MPD1 and MAH1) in *T. kodakarensis* was performed as described previously (Sato et al., 2003, 2005; Hirata et al., 2008). The sequences of all PCR primers used for this study are listed in Table 2. For Mbh overexpression in *T. kodakarensis*, four vectors (pMHG1, pMHC1, pMPD1, and pMAH1) were constructed as follows. Schemes of the cloning strategies are shown in the Supplementary Materials, Figure S1 for construction of pMHG1, Figure S2 for pMHC1, Figure S3 for pMPD1 and Figure S4 for pMAH1.

**Table 2:** **Sequences of primers used in this study**.

**Plasmid used for**	**Name**	**Sequence (from 3′ to 5′)**
pUP1	PyrF-N-SP	AAAAACTAGTCCGCAACGCGCATTTTGCTCACCC
pUP1	M13RV	CAGGAAACAGCTATGAC
pUP1	2μm-Sp	AAAAACTAGTGATAAGCTGTCAAAGATGAG
pUP1	2μm-Xb	AAAATCTAGAATGCGACGTGCAAGATTACC
pMHG1	gdh-Nd	AAAACATATGTACCACCTCATTTCGGTAATCTGCGAGG
pMHG1	gdh-Xb	AAAATCTAGATATCCCACCTCCGATTCCGTTGG
pMHG1	mhp1	AAAAGAATTCGGCTGGAGCGTTCATCGCCTTCG
pMHG1	mhp2	AAAATCTAGAGCTTAAAACGCTTTTCCCAAGC
pMHG1	mhp3-3	AAAATCTAGAAAAAACATATGTTGCCGTTCATAGTGGCGTTCCTC
pMHG1	mhp4	AAAAGTCGACCCTCGTAGGCATCAACAACCGC
pMHC1	Tk-mbhJ-Nh	AAAAGCTAGCATGGCGATAACAGTTCCCGCCAAC
pMHC1	Tk-mbhN-Bm	AAAAGGATCCACCTACGGTGAAGAACCGAAAAAA
pMHP1	mhpd1	AAAGGATCCAACCCTCATAGTAGGCAACGCGA
pMHP1	mhpd4	AAAGAATTCAGGCGGAGCGGGTAGATGCCCTC
pMHP1	mhpd2-2	AAACCCTTCATCCCCATATCA
pMHP1	mhpd3-2	CAAAAACACACTCTGCGGAGGTGGTAGCTGATG
pMAH1	csgx	AAAATCTAGACGGCAAAAGGCGAATTATGTG
pMAH1	csgn	AAAACATATGACAACACCTCCTTGGGTTG

#### Construction of pMHG1

pUP1 is a plasmid that contains the *pyrF* marker gene of *T. kodakarensis* flanked by identical sequences (2 μm), necessary for marker removal *via* homologous recombination after cloning. pUP1 was constructed by amplification of the *pyrF* region from the pUD plasmid (Sato et al., 2003) with the primer set, PyrF-N-SP/M13RV and inserting the fragment into the SpeI and XbaI sites of pUC19-Sp. pUC19-Sp is a modified pUC19 plasmid containing an SpeI recognition site instead of the SmaI recognition site. Next, the primer set 2 μm-Sp/2 μm-Xb was used to amplify the 2 μm region from the yeast expression vector pYES (Life Technologies, Carlsbad, CA), and the fragment was inserted into the SpeI site upstream of the *pyrF* gene, and again into the XbaI site downstream of the *pyrF* gene. To enable further cloning *via* NdeI, an NdeI site (CATATG) inside of the *pyrF* gene of pUP1 was changed to CACATG by point mutation (underline indicates the position of the changed nucleotide), resulting in plasmid pUP1m. The promoter region of the glutamate dehydrogenase gene (TK1431) (P_*gdh*_) was amplified from the genomic DNA of *T. kodakarensis* using the primer set gdh-Xb/gdh-Nd. The amplified fragment was inserted into the SpeI/NdeI site of pUP1m to yield plasmid pUPG1. Two genomic regions (1.0–1.1 kb each) including the promoter of the *mbh* operon (P_*mbh*_) and a part of the *mbh* structural genes (*mbhA*) were amplified with the primer sets mhp1/mhp2 and mhp3-3/mhp4, respectively. The resulting fragments were cut by EcoRI/XbaI and XbaI/SalI, respectively, and were fused and inserted into the EcoRI/SalI sites of pUC118, resulting in plasmid pMHGa. A point mutation (T to C) was introduced to the 204th nucleotide of *mbhA*, to change an existing NdeI site (CATATG) to CATACG (underline indicates the position of the changed nucleotide), yielding plasmid pMHGam. The point mutation resulted in a change of the respective (68th) codon from TAT to TAC, both encoding the same amino acid (tyrosine). Next, a fragment containing 2 μm-*pyrF*−2 μm-P_*gdh*_ was cut from pUPG1 by NdeI/XbaI and introduced to the respective sites of pMHGam, to obtain plasmid pMHG1.

#### Construction of pMHC1

First, the *mbhJKLMN* genes as well as its terminator region and the cell surface glycoprotein gene (TK0895) promoter (P_*csg*_) were amplified from the genomic DNA of *T. kodakarensis* by PCR using the primer sets Tk-mbhJ-Nh/Tk-mbhN-Bm and Pcsg-Sp/Pcsg-Nh, respectively. *Via* the introduced SpeI, NheI, and BamHI cleavage sites of these fragments, P_*csg*_ and *mbhJKLMN* were fused and inserted into the SpeI/BamHI sites of pUC19-Sp, yielding the plasmid pMH1. Second, SpeI and XbaI were used to clone the promoter gene cassette into the XbaI site of plasmid pUP1 containing the 2 μm-*pyrF*−2 μm cassette, resulting in plasmid pMHUP1. In the third step, the cassette including P_*csg*_, *mbhJKLMN* and 2 μm-*pyrF*−2 μm was excised *via* SpeI and inserted into the SpeI site of the plasmid pchiA-NC, to yield plasmid pMHC1. pchiA-NC is a pUC118 derivative with 0.9–1.0 kb homologous sequences of the 5′-flanking region of the *T. kodakarensis* chitinase gene (*chiA*,TK1765) and of the 3′-portion of the gene itself. After amplification of the 5′-flanking region and the 3′-portion of *chiA* from the genome using primer sets ChiA-1/ChiA-2 and ChiA-3/ChiA-4, respectively, both fragments contained overlapping regions upstream of the introduced SpeI sites and were fused in a second fusion PCR reaction using the primer set ChiA-1/ChiA-4. The resulting fragment was inserted into the EcoRI and SalI sites of the multi-cloning site of pUC118 upon digestion and blunt ending to yield pchiA-NC. The final plasmid pMHC1 carries the *mbhJKLMN* genes with its terminator region (T_mbh_) and the 2 μm-*pyrF*−2 μm cassette, flanked by the *chiA* sequences for homologous recombination.

#### Construction of pMPD1

A palindrome sequence (5′-TCCGCGAGAGCTCTGCGGA-3′) is located within a non-coding region (37 bp) between the Mbh subunit structure genes *mbhI* and *mbhJ*. The non-coding region was amplified together with its adjacent *mbh* genes from the genomic DNA of *T. kodakarensis* using the primer set mhpd1/mhpd4. The fragment was cut by BamHI/EcoRI, and ligated into the respective sites of plasmid pUD2 (Sato et al., 2005), to yield plasmid pMPDa. In order to disrupt the palindrome sequence on pMPDa *via* nucleotide substitution (5′-CAAAAACACACTCTGCGGA-3′; underline indicates the positions of mutated nucleotides), inverse PCR was performed using the primer set mhpd2-2/mhpd3-2, and the amplified fragment was self-ligated to obtain plasmid pMPD1.

#### Construction of pMAH1

Using genomic DNA of *T. kodakarensis*, P_*csg*_ was amplified with the primer set csgx/csgn, and the fragment cut by XbaI/NdeI was introduced to the respective sites of pMHGam, to yield plasmid pMAHa. A fragment containing the P_*mbh*_ region, P_*csg*_ and a part of the *mbhA* structure gene was excised from this plasmid by sequentially applying SalI, DNA blunting and EcoRI digestion. The fragment was introduced into the EcoRI/SmaI site of pUD2, to obtain plasmid pMAH1.

DNA restriction and modification enzymes as well as general cloning plasmids were purchased from TaKaRa (Otsu, Japan) or Toyobo (Osaka, Japan). The KOD plus NEO DNA polymerase (Toyobo) was used for amplification, and DNA fragments separated *via* agarose gel electrophoresis were isolated using the MinElute gel extraction kit (Qiagen, Hilden, Germany). Plasmids were isolated with the Plasmid Mini kit (Qiagen). The cloning products were confirmed *via* sequencing with the BigDye Terminator cycle sequencing kit, version 3.1 and a model 3130 capillary DNA sequencer (Applied Biosystems, Foster City, CA).

For transformation, the *T. kodakarensis* uracil-auxotroph strains KU216 (Sato et al., 2005) (for MHG1, MHC1, and MPD1) and DPHA1 (Kanai et al., 2011) (for MAH1) were used as host strains. The transformation procedures included selection of *pyrF*^+^ strains with uracil-prototrophy and positive selection of *pyrF*-eliminated strains with 5-fluoroorotic acid and was performed as described elsewhere (Sato et al., 2005; Hirata et al., 2008; Kanai et al., 2011). Recombinant strains carrying the desired genetic modifications on the genome were identified by colony PCR and sequencing.

### Western blot analysis

To determine intracellular protein levels of MbhL, Western blot analysis was performed. *T. kodakarensis* strains (KU216, DPHA1, MHG1, MHC1, MPD1, and MAH1) were cultivated in MA-YT medium supplemented with 0.5% (w/v) sodium pyruvate. After 11 h of cultivation at 85°C, cells were harvested by centrifugation under 5000 *g* for 10 min at 4°C. Cell pellets were resuspended in 25 mM Tris-HCl (pH 8.0) buffer containing 0.1% (v/v) Triton-X100, and disrupted by vortex for 30 min at 4°C. After removing the insoluble fraction by centrifugation under 5000 *g* for 10 min, the resulting cell extracts were used for Western blot analysis. Protein concentrations were measured using the Bio-Rad protein assay kit (Bio-Rad Laboratories, Hercules, CA), with bovine serum albumin as the standard. Sodium dodecyl sulfate-polyacrylamide gel electrophoresis (SDS-PAGE) was performed in a 12.5% gel. Western blot analysis was performed as described previously (Endoh et al., 2006) using rabbit polyclonal antibodies against the MbhL protein.

### Continuous culture experiments

Continuous culture experiments of the host strains KU216 and DPHA1 and the engineered strains MHG1, MHC1, MPD1, and MAH1 were performed as described previously (Kanai et al., 2011) using a gas-lift fermenter designed for cultivation of hyperthermophiles (Taiyo Nippon Sanso Corporation, Tokyo, Japan). In a 1 L cultivation vessel, 500 mL of MA-YT-Pyr medium was introduced and cultivation was performed at 85°C with continuous agitation using a rotor at 50 rpm. The evolved gas metabolites were flushed out by nitrogen gas, which was introduced continuously into the vessel at a rate of 100 mL min^−1^. Fresh medium was supplied into the vessel using a peristaltic pump and the volume of the culture was monitored with a water level sensor (B.E. Marubishi, Tokyo, Japan), which was connected to a pump for culture discharging. Cell densities were monitored by measuring the turbidity at 660 nm (OD_660_) and according biomasses (dcw) were calculated from OD_660_ *via* calibration information determined beforehand. The pH of the culture broth was maintained at 7.4 and the amounts of H_2_ gas and CO_2_ gas in the exhaust gas were measured periodically using gas chromatography (provided by Taiyo Nippon Sanso Corporation) as described previously (Kanai et al., 2005).

## Results

### Construction of *T. kodakarensis* strains that overexpress the Mbh genes

In *T. kodakarensis*, the membrane-bound hydrogenase, Mbh, is the key enzyme that is responsible for the evolution of H_2_ (Kanai et al., 2011). The *mbh* operon can be divided into two regions; the former region containing genes presumed to encode Na^+^/H^+^ antiporter subunits (*Na*/*H* region; *mbhA-I*; TK2080-TK2088), and the latter region containing genes for the catalytic [NiFe]-hydrogenase subunits (*Hyd* region; *mbhJ-N*; TK2089-TK2093) (Figure 2). These two regions are separated by a palindrome sequence (5′-TCCGCGAGAGCTCTGCGGA-3′) that can form a remarkably long stem-loop structure and may potentially inhibit transcription and/or translation.

**Figure 3:**
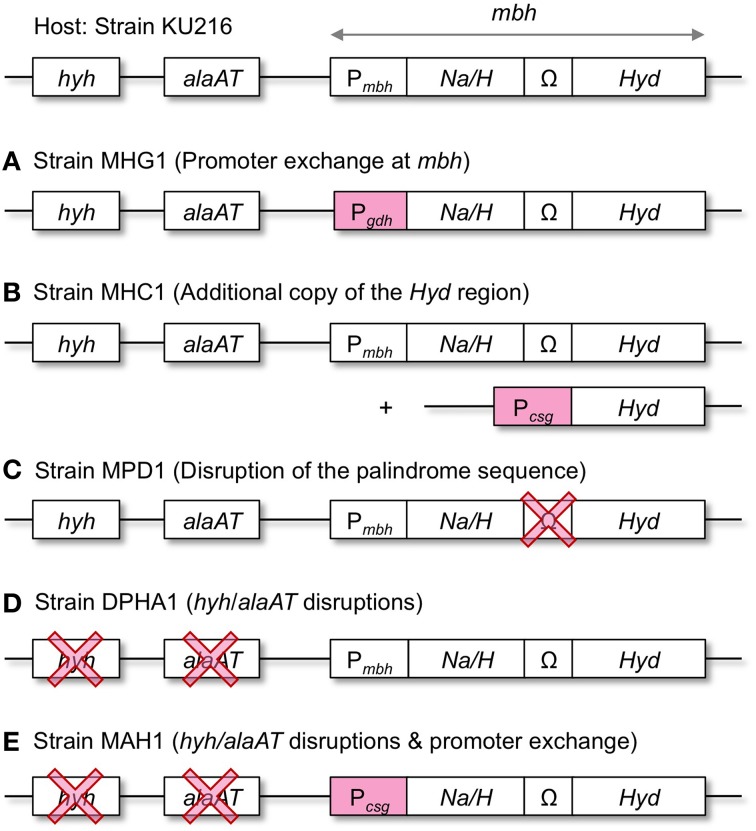
**Overview of the genetic approaches taken to increase H_2_ production in *T. kodakarensis*. (A–C)**, Mbh overexpression; **(D)**, Disruption of the pyruvate reduction pathway associated with H_2_ consumption; **(E)**, Combination of both approaches. *hyh*, cytosolic [NiFe]-hydrogenase gene; *alaAT*, alanine aminotransferase gene; *Na/H*, Na^+^/H^+^-antiporter region of Mbh (*mbhA-I*); Ω, palindromic sequence; *Hyd*, [NiFe]-hydrogenase catalytic region of Mbh (*mbhJ-N*); P_*mbh*_, *mbh* promoter; P_*gdh*_, *gdh* promoter; P_*csg*_, *csg* promoter.

In order to enhance the capacity of H_2_ production, we took three different genetic approaches aiming to increase the Mbh protein levels in *T. kodakarensis* (Figure 3). First, the *mbh* promoter (P_*mbh*_) of the entire operon was exchanged with the strong/constitutive glutamate dehydrogenase gene (TK1431) promoter (P_*gdh*_) (strain MHG1). Second, the *Hyd* region, which encodes the catalytic subunits, was overexpressed under the control of another strong/constitutive cell-surface glycoprotein gene (TK0895) promoter (P_*csg*_) (strain MHC1). The construct was inserted into the *chiA*-locus, which encodes a chitinase (Tanaka et al., 1999), resulting in a strain with a second copy of the *Hyd* region. Third, the palindrome sequence between *mbhI* and *mbhJ* was deleted, as the *Hyd* gene cluster falls downstream of the palindrome, and removal of the sequence might enhance the expression of the *Hyd* genes (strain MPD1). All modifications were introduced into the genome of *T. kodakarensis* strain KU216 by homologous recombination and were confirmed *via* analytical PCR and sequencing (data not shown).

### Quantification of MbhL protein in the recombinant strains

**Figure 4:**
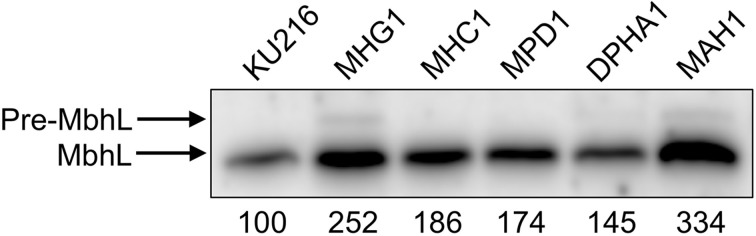
**Expression levels of the [NiFe]-hydrogenase large subunit (MbhL) in the constructed *T. kodakarensis* strains**. Numbers below the panel indicate band intensity (%) relative to that of the host strain KU216 (defined as 100%). Pre-MbhL indicates the MbhL precursor (see Discussion).

In order to compare the Mbh production levels of the constructed *T. kodakarensis* strains, Western blot analysis was performed on the extracts of cells grown in pyruvate medium (MA-YT-Pyr) and compared (Figure 4). Antibodies raised against the large subunit of Mbh (MbhL) were applied to estimate the overexpression of the catalytic Hyd subunits.

Quantification of the bands revealed that protein levels of MbhL were higher in all three recombinant strains compared to that observed in the host strain KU216. MbhL levels in strain MHC1 (addition of Mbh *Hyd* genes under the control of P_*csg*_) and strain MPD1 (deletion of the palindrome sequence) increased 1.86-fold and 1.74-fold, respectively. Strain MHG1, whose *mbh* operon is under the control of P_*gdh*_, displayed even higher levels of MbhL, 2.52-fold higher than that of the host strain. Extracts from this strain revealed an additional band (Pre-MbhL) with a higher molecular weight than that of MbhL (see Discussion).

### Hydrogen production under continuous culture conditions

HERs of the Mbh overexpression strains (MHG1, MHC1, and MPD1) were examined and compared with that of the host strain (KU216). If the H_2_-forming Mbh reaction is the bottleneck of H_2_ production from pyruvate, increases in MbhL protein might result in increases in SHPR. To investigate this relationship, cell- and culture volume-specific H_2_ production rates (SHPR, HER) of the *T. kodakarensis* strains were analyzed under continuous culture conditions using a continuous gas-flow fermenter.

At a dilution rate of 0.27–0.31 h^−1^, cell densities (OD_660_) of all strains were between 0.84 and 1.09 (Table 3). In these cultures, HERs ranged from 9.4 to 11.2 mmol L^−1^ h^−1^ with the host strain KU216 displaying the lowest H_2_ production, while the highest production was observed with strain MHC1.

**Table 3:** **Average cell densities, H_2_ productivities (HERs and SHPRs), and molecular H_2_/CO_2_ ratios of *T. kodakarensis* strains**.

**Strain**	***D* (h^−1^)**	**OD_660_**	**HER (mmol L^−1^ h^−1^)**	**SHPR (mmol g-dcw^−1^ h^−1^)**	**H_2_/CO_2_**
KU216	0.27	0.95 ± 0.01	9.4 ± 0.3	28.3 ± 0.9	0.96
MHG1	0.31	0.84 ± 0.01	10.3 ± 0.1	35.3 ± 0.5	0.91
MHC1	0.27	0.98 ± 0.01	11.2 ± 0.3	32.4 ± 1.2	0.93
MPD1	0.30	1.09 ± 0.01	11.1 ± 0.6	28.9 ± 1.4	0.88
DPHA1	0.30	1.15 ± 0.01	11.7 ± 0.5	28.7 ± 1.4	0.96
MAH1	0.30	1.02 ± 0.01	13.0 ± 0.3	36.2 ± 1.1	0.98
MAH1	0.59	0.91 ± 0.03	24.6 ± 0.6	76.7 ± 2.4	0.99
MAH1	0.83	0.64 ± 0.03	27.1 ± 1.6	120 ± 2	1.04
MAH1	1.07	0.41 ± 0.02	17.4 ± 0.9	120 ± 9	1.18

D, Dilution rate; Error bars represent standard deviations of at least three measured points at the steady state of each dilution rate.

As the HER depends on the cell densities, differences in SHPR more accurately reflect the impact of genetic modification on H_2_ production in the cell. The deletion of a palindrome sequence in strain MPD1 caused an increase in MbhL protein (Figure 4), but hardly changed the SHPR (Table 3). For the other strains, on the other hand, there was a general tendency that strains with higher levels of MbhL protein resulted in higher SHPR values; 28.3 (host), 32.4 (strain MHC1) and 35.3 mmol g-dcw^−1^ h^−1^ (strain MHG1).

### Effect of combining Mbh overexpression with deletion of the pyruvate reduction pathway linked to H_2_ consumption

Promoter exchange by P_*gdh*_ (strain MHG1) exhibited the highest effect among the three Mbh overexpression strains examined. As a next step, we focused on the disruption of the pyruvate reduction pathway to alanine. The pathway is metabolically linked to H_2_ consumption and its disruption circumvents H_2_ uptake of *T. kodakarensis* (Kanai et al., 2011). The double knock out strain (DPHA1) carries *hyh* and *alaAT* gene deletions and was previously shown to exhibit a higher SHPR than its host strain KU216 (Kanai et al., 2011). To check whether Mbh overexpression and deletion of *hyh* and *alaAT* have an additive effect on H_2_ production, DPHA1 was further engineered to overexpress the *mbh* operon *via* promoter exchange with P_*csg*_, resulting in strain MAH1.

Levels of MbhL protein in strain DPHA1 and in strain MAH1 were examined *via* Western blot analysis using anti-MbhL antibodies. As a result, MAH1 exhibited strikingly higher levels of MbhL; 3.34-fold and 2.30-fold higher band intensities were observed when compared to those of the strains KU216 and DPHA1, respectively (Figure 4). The results also indicate that the MbhL protein levels in MAH1 are higher than those in MHG1, and as such, intracellular Pre-MbhL accumulation found in MHG1 was also observed in strain MAH1 (see Discussion).

Evaluations of HERs in continuous cultures of DPHA1 and MAH1 were examined at a dilution rate of 0.30 h^−1^. Unlike the previously reported examination (Kanai et al., 2011), *hyh* and *alaAT* deletion (strain DPHA1) only slightly increased SHPR (Table 3). In contrast, strain MAH1 exhibited the highest increases in SHPR with 36.2 mmol g-dcw^−1^ h^−1^. The increase of SHPR by 28% is slightly above the increase caused by the promoter exchange with P_*gdh*_ in strain MHG1 (25%). This agrees with the higher MbhL protein levels found in strain MAH1 than in MHG1. The higher levels of MbhL in MAH1 compared to those in MHG1 may be due to differences in the strengths of the promoters P_*csg*_ and P_*gdh*_. However, the additional disruption of *hyh* and *alaAT* in MAH1 may also have an effect, as the MbhL levels in DPHA1 are higher than those in KU216, even though there are no changes in the promoters governing *mbhL* expression. In addition to the high SHPR, strain MAH1 also exhibited the highest HER (13.0 mmol L^−1^ h^−1^) among the strains examined at a dilution rate of around 0.3 h^−1^.

### Influence of the culture dilution rates on SHPRs

**Figure 5:**
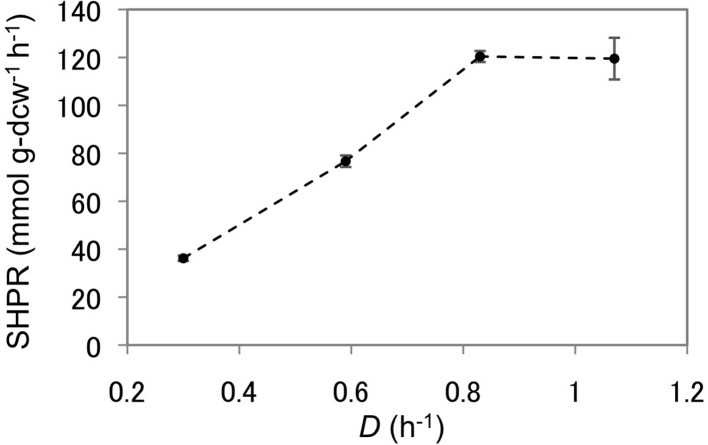
**SHPRs of strain MAH1 at different dilution rates**. Error bars represent standard deviations of at least three measured points during the steady-state of each dilution rate. *D*, Dilution rate.

As *T. kodakarensis* strain MAH1 displayed the highest SHPRs and HERs, this strain was used to analyze the effect of dilution rates on H_2_ production from pyruvate. The dilution rate was increased stepwise from 0.30 to 0.59, 0.83, and 1.07 h^−1^. SHPRs as well as HERs increased gradually and both displayed their maxima at a dilution rate of 0.83 (Figure 5, Table 3). The SHPR and HER at this dilution rate were 120 mmol g-dcw^−1^ h^−1^ and 27.1 mmol L^−1^ h^−1^, respectively. Both values (SHPR and HER) are so far the highest of those reported for *T. kodakarensis*. At a dilution rate of 1.07 h^−1^, SHPR maintained a constant value of 120 mmol g-dcw^−1^, whereas the volume-specific HERs dropped to 17.4 mmol L^−1^ h^−1^ as a result of a decrease in cell density.

## Discussion

In this study, different strategies were taken to overproduce the [NiFe]-hydrogenase complex Mbh in *T. kodakarensis* and to reduce H_2_-consuming pathways. The H_2_ production potential of these engineered strains were examined in a continuous culture, where evaluation is possible under steady-state conditions. As a result, we found that the increase in SHPR was highest in strain MAH1, with a 28% increase compared to the host strain at dilution rates of 0.27–0.31 h^−1^.

In comparison, the SHPR from formate in *E. coli* increased by 2.8-fold in a batch culture when deleting a negative transcription regulator and overexpressing a transcriptional activator of the formate hydrogenlyase complex (strain SR13 in Table 4) (Yoshida et al., 2005). In *T. onnurineus* KS0413, also in a batch culture, up to 2.9-fold increased SHPRs were reached by promoter exchange of the carbon monoxide dehydrogenase (CODH) operon including CODH, hydrogenase and an Na^+^/H^+^ antiporter with P_*csg*_ (Table 4) (Kim et al., 2013; Lee et al., 2014). In both cases, hydrogenase overexpression yielded much higher increases in SHPR compared to those obtained in this study. This is most likely due to the fact that the substrate to H_2_ conversion (formate –> H_2_+ CO_2_ or CO + H_2_O –> CO_2_+ H_2_) comprises only one enzymatic step which was subjected to overexpression. In contrast, the H_2_ production from pyruvate in *T. kodakarensis* involves at least one additional enzyme, POR, and the flux might also be affected by the downstream ACS (Figure 1). As we did observe 25–28% increases in SHPR in strains MHG1 and MAH1, the Mbh reaction seems to be the rate-limiting step for H_2_ production from pyruvate in the wild type *T. kodakarensis*. The maximal increase in SHPR upon Mbh overexpression was probably reached, as promoter exchange of P_*mbh*_ with P_*csg*_ provided higher protein levels (334%, strain MAH1) than P_*gdh*_ (252% strain MHG1), but only slightly increased SHPR values (36 compared to 35 mmol g-dcw^−1^ h^−1^). In order to reach higher SHPR values, a simultaneous increase in the levels of Mbh, POR, and ACS may be necessary.

**Table 4:** **Strong microbial H_2_ producers and their maximal H_2_ production rates**.

	**Organism**	**Substrate**	**Culture conditions**	**SHPR**	**HER**	**Reference**
Continuous culture	*Thermococcus kodakarensis* MAH1	Pyruvate	Gas removal, *D*: 0.83, *T*: 85	120.4	27.1	This study
	*Pyrococcus furiosus* DSM3638	Maltose	*D*: 0.6, *T*: 98	102^*^	–	Schicho et al., 1993
	*Thermococcus kodakarensis* KOD1	Pyruvate	Gas removal, *D*: 0.8, *T*: 85	59.6	6.3	Kanai et al., 2005
	*Clostridium sp*. No. 2	Glucose/Xylose	*D*: 1.2-1.3, *T*: 36	34.0/41.9	20.4/15.1	Taguchi et al., 1995
	*Caldicellulosiruptor kristjanssonii* DSM12137	Glucose	*D*: 0.15, *T*: 70	34.6	10.3	Zeidan et al., 2010
	*Klebsiella oxytoca* HP1	Sucrose	*T*: 38	15.2	14.4^#^	Minnan et al., 2005
Batch culture	*Thermococcus onnurineus* NA1	Formate	*T*: 80	351.6	85.8	Bae et al., 2015
	*Escherichia coli* SR13	Formate	Enriched cells in buffer, substrate feed, *T*: 37	250.0	12,351.3^#^	Yoshida et al., 2005
	*Thermococcus onnurineus* KS0413	CO	pH control, CO feed, *T*: 80	207.8	88.4	Lee et al., 2014
	*Citrobacter sp.* Y19	Glucose	*T*: 36	32.3	4.9^#^	Oh et al., 2003
	*Enterobacter cloacae* IIT-BT 08	Sucrose	pH control, *T*: 36	29.5	35.6	Kumar and Das, 2000
	*Ethanoligenens harbinense* B49	Glucose	*T*: 36	27.7	7.5^*^	Xu et al., 2008
	*Klebsiella oxytoca* HP1	Glucose	In buffer, *T*: 35	9.6	3.6^#^	Minnan et al., 2005
	*Thermoanaerobacterium thermosaccharolyticum* W16	Glucose/Xylose	*T*: 60	9.7/8.8	12.9/10.7	Ren et al., 2008
	*Thermotoga elfii* DSM9442	Glucose	*T*: 65	8.9	4.5	van Niel et al., 2002
	*Thermotoga neapolitana* DSM4359	Xylose	*T*: 80	0.24	1.45	Eriksen et al., 2011
	Photosynthetic bacteria and algae	Organic acids, sugars	*T*: 35	< 6	< 6	Hillmer and Gest, 1977

SHPR (mmol g-dcw^−1^ h^−1^); HER (mmol L^−1^ h^−1^); D, Dilution rate (h^−1^); T, cultivation temperature (°C);

* Values estimated from a plot;

# Converted from ml/L/h via gas constant at 23°C and 1 atm.

Interestingly, we observed the presence of the precursor of the large Mbh subunit (Pre-MbhL) at high Mbh overexpression levels (strains MHG1 and MAH1 in Figure 4). Posttranslational maturation of the active center of the large Mbh subunit (MbhL) is assisted by the Mbh accessory Hyp proteins (Sasaki et al., 2012, 2013; Watanabe et al., 2012a, 2015; Tominaga et al., 2013), which is completed by the cleavage of the Pre-MbhL protein into the functional MbhL *via* specific endopeptidases (Forzi and Sawers, 2007; Watanabe et al., 2012b). The increased levels of Pre-MbhL in strains MHG1 and MAH1 may be exceeding the functional capacity of the Hyp proteins, thereby leading to the accumulation of precursor. The Na^+^/H^+^ antiporter does not seem to be required for Mbh maturation, as overexpression of the Hyd region without the Na/H region in strain MHC1 resulted in an increase in mature MbhL (Figure 4) and increased H_2_ production (Table 3).

The increase in SHPR brought about by deletion of *hyh* and *alaAT* in this study was lower than those observed elsewhere (Kanai et al., 2011). In batch cultures, three-fold higher cell specific H_2_ productions from pyruvate were reached when *hyh* was disrupted and an estimated 9% higher H_2_ productions when *alaAT* was disrupted (Santangelo et al., 2011). The continuous removal of H_2_ from the gas phase in our cultures is probably the reason for the much lower effects of *hyh* and *alaAT* disruption on H_2_ consumption. Large effects of gas removal on H_2_ production (54% increase) have also been demonstrated in studies with a mixed microbial culture and glucose as a substrate. Gas removal was suggested to prevent H_2_ (and CO_2_) consumption by homoacetogenesis (Esquivel-Elizondo et al., 2014). Increased H_2_ concentrations in the liquid phase caused by higher gas phase pressures were also assumed to influence the equilibrium of the H_2_ production step in *E. cloacae* (Mandal et al., 2006).

Among fermentative microorganisms, the *T. kodakarensis* strain MAH1 exhibits relatively high H_2_ microbial production rates (Table 4). This demonstrates the high potential of this strain as a host strain for further engineering. Examining the H_2_ production of this strain grown on cheaper substrates like sugars will be important, as demonstrated with *P. furiosus* (Schicho et al., 1993), which is also a strong H_2_ producer. The HER can probably be further enhanced by increasing cell densities, for example by cell immobilization (Zhao et al., 2012). Studies with the *E. coli* strain SR13 showed that beside genetic modification, the use of concentrated cells results in extremely high H_2_ yields (Yoshida et al., 2005).

### Conflict of interest statement

The authors declare that the research was conducted in the absence of any commercial or financial relationships that could be construed as a potential conflict of interest.

## Supplementary material

Figure S1 **Strategy for construction of pMHG1**. pMHG1 was used to insert P*_gdh_* upstream of the *mbhA* gene of the *mbh* operon *via* homologous recombination using strain KU216 as the host.

Figure S2 **Strategy for construction of pMHC1**. pMHC1 was used to introduce an additional *Hyd* gene region under the control of P*_csg_* into the chitinase region of strain KU216 *via* homologous recombination.

Figure S3 **Strategy for construction of pMPD1**. pMPD1 was used to replace the palindrome sequence between the *Na/H*- and *Hyd* regions of strain KU216 with a non-coding sequence that does not form a stem loop structure *via* homologous recombination.

Figure S4 **Strategy for construction of pMAH1**. pMAH1 was used to introduce P*_csg_* upstream of the *mbhA* gene of the *mbh* operon *via* homologous recombination. Strain DPHA1 was used as the host in order to combine Mbh overexpression with *alaAT* and *hyh* deletion.
