# Supplementary figures and images for: Overproduction of the membrane-bound [NiFe]-hydrogenase in Thermococcus kodakarensis and its effect on hydrogen production
Source: Front Microbiol. 2015 Aug 26;6:847. doi: 10.3389/fmicb.2015.00847 (PMC4549637; doi:10.3389/fmicb.2015.00847)

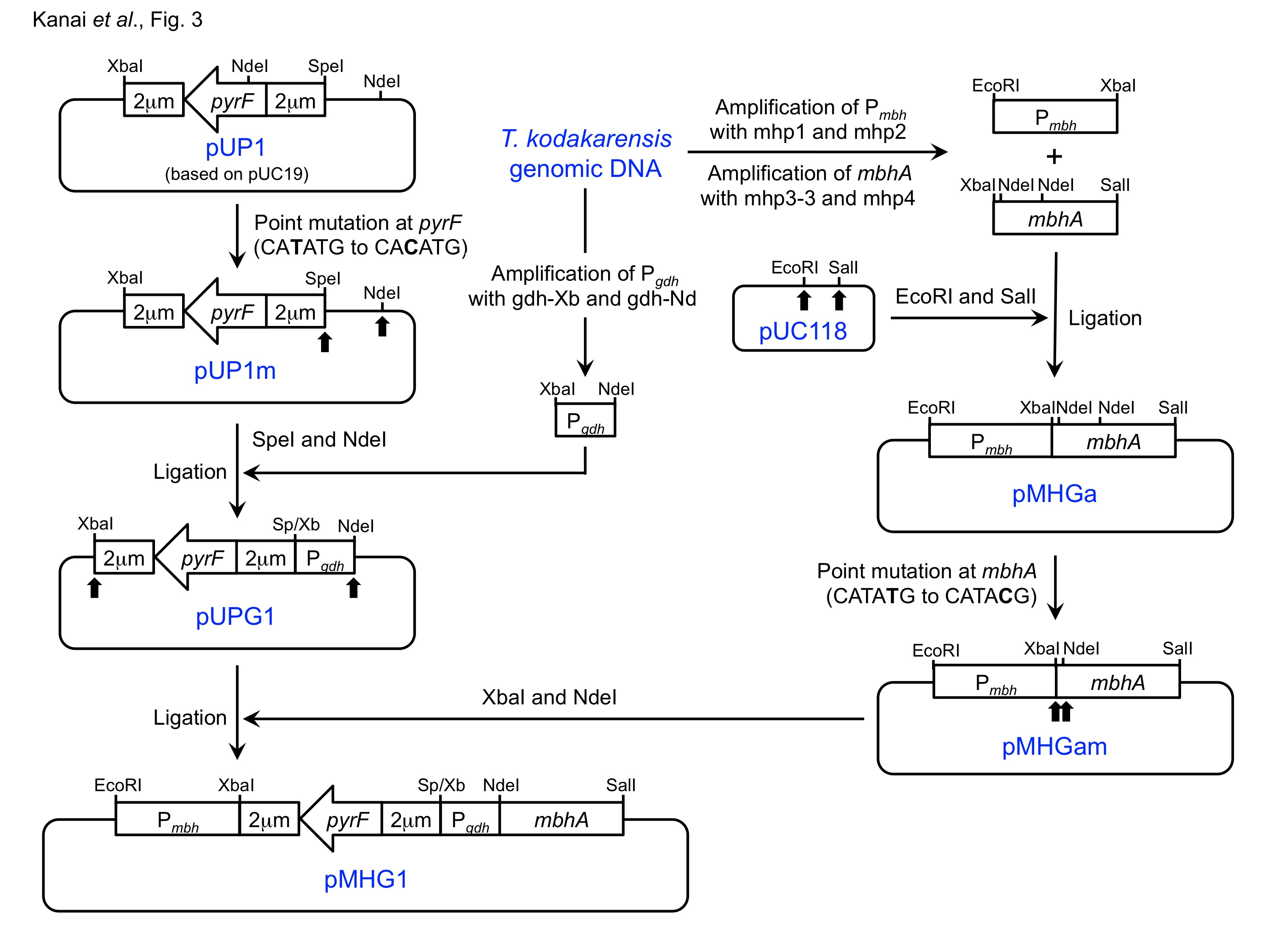

Supplement: Figure S1 — Strategy for construction of pMHG1. pMHG1 was used to insert Pgdh upstream of the mbhA gene of the mbh operon via homologous recombination using strain KU216 as the host. [file Figure1.JPEG]

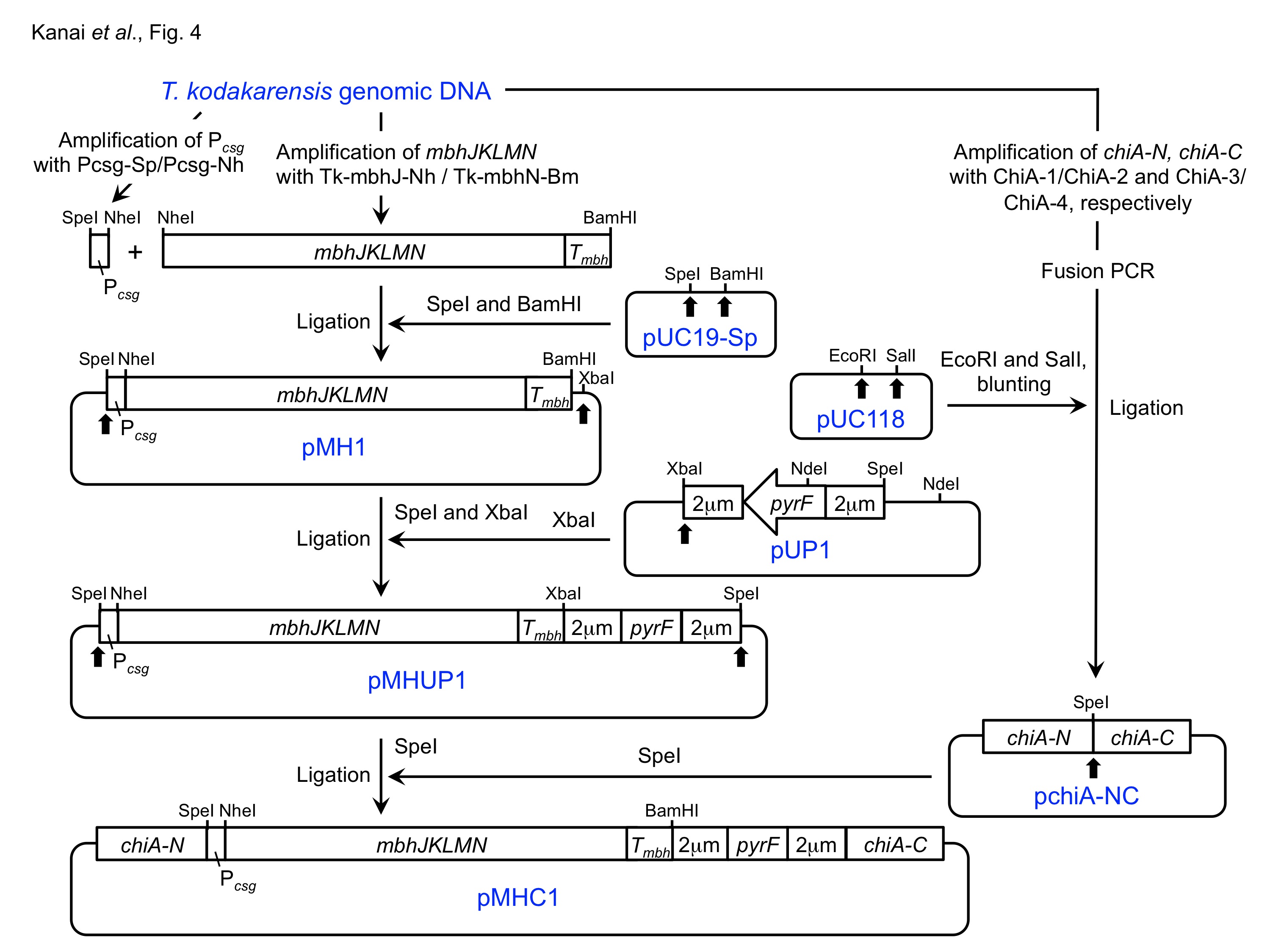

Supplement: Figure S2 — Strategy for construction of pMHC1. pMHC1 was used to introduce an additional Hyd gene region under the control of Pcsg into the chitinase region of strain KU216 via homologous recombination. [file Figure2.JPEG]

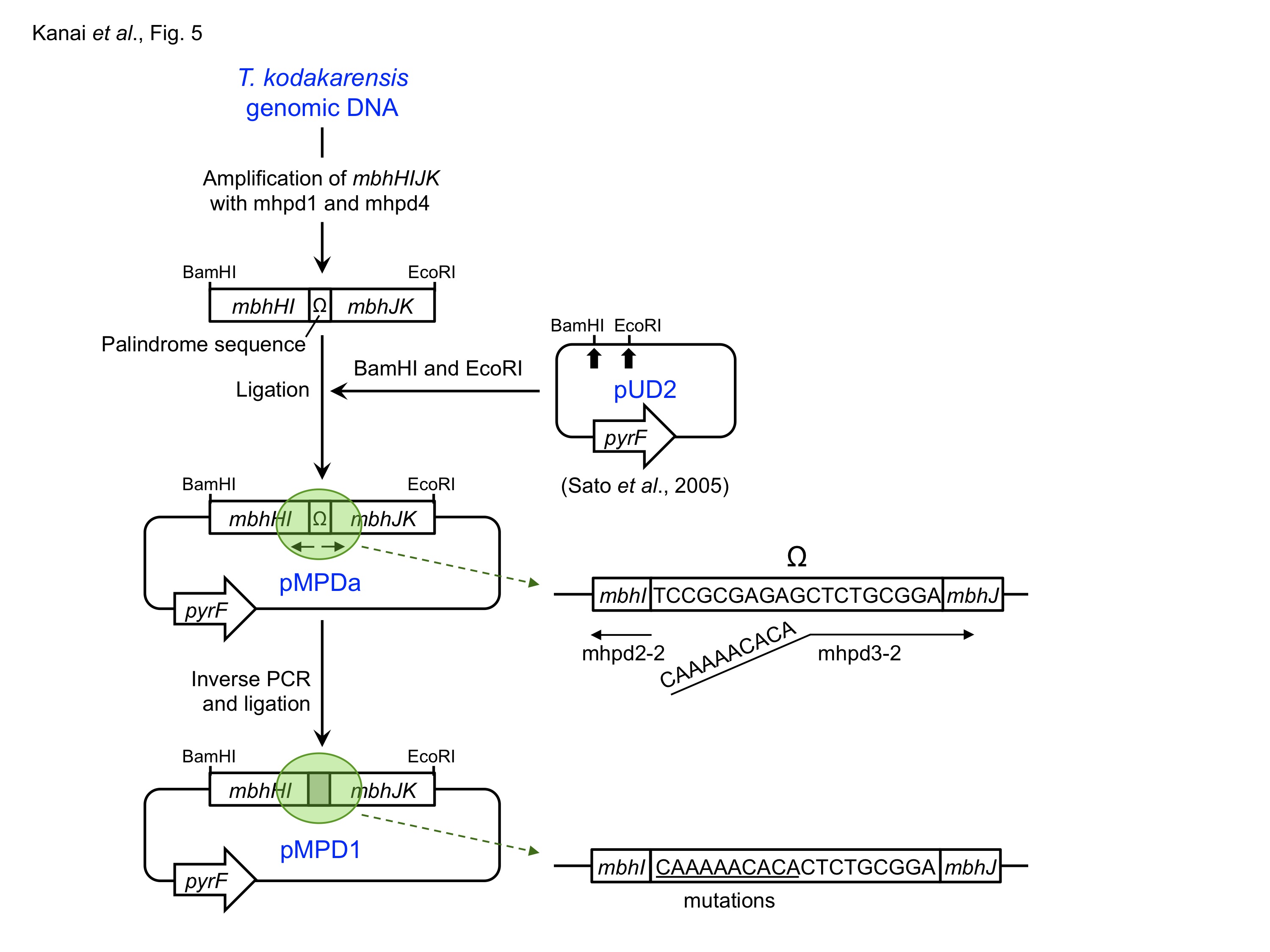

Supplement: Figure S3 — Strategy for construction of pMPD1. pMPD1 was used to replace the palindrome sequence between the Na/H- and Hyd regions of strain KU216 with a non-coding sequence that does not form a stem loop structure via homologous recombination. [file Figure3.JPEG]

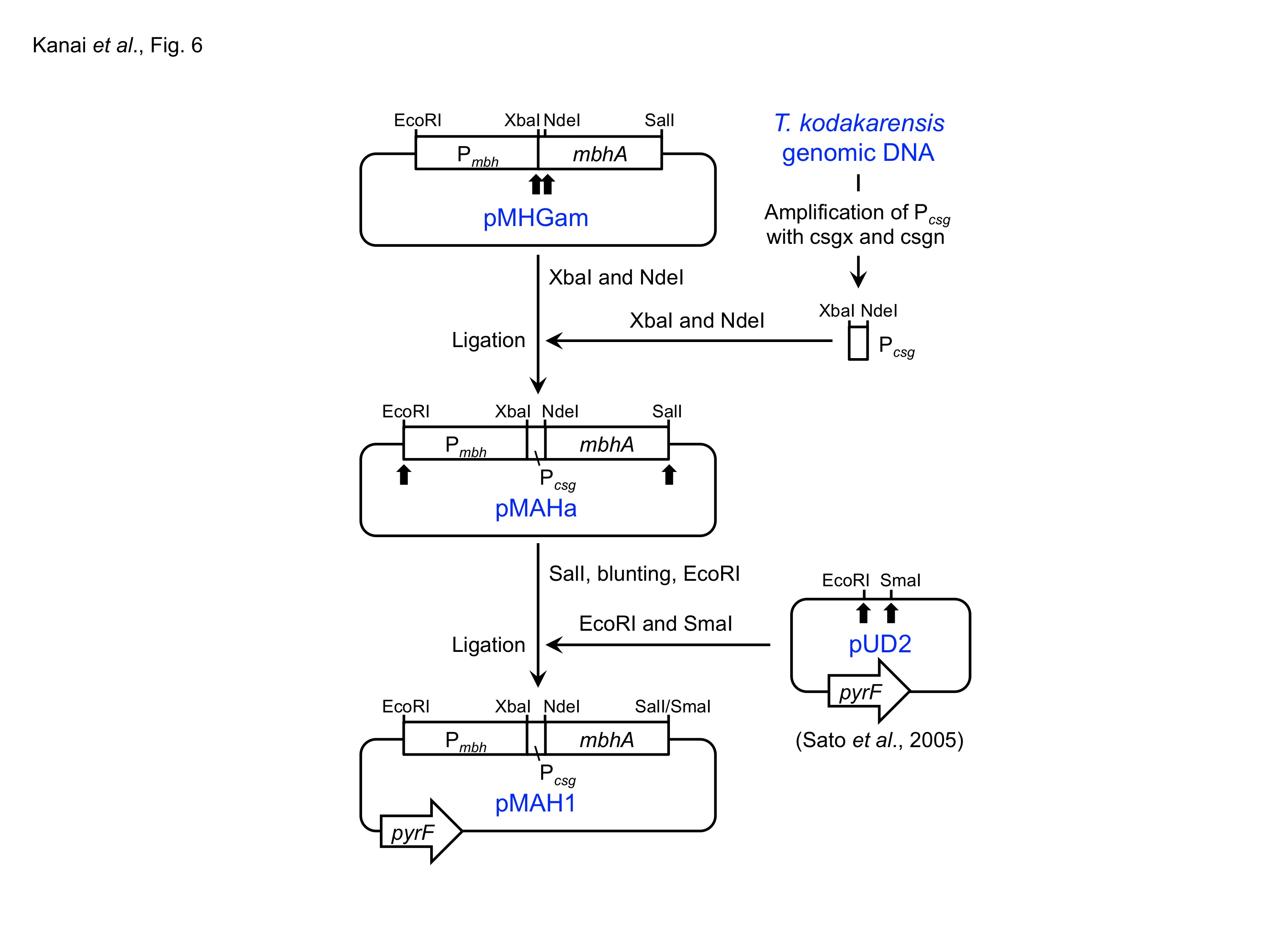

Supplement: Figure S4 — Strategy for construction of pMAH1. pMAH1 was used to introduce Pcsg upstream of the mbhA gene of the mbh operon via homologous recombination. Strain DPHA1 was used as the host in order to combine Mbh overexpression with alaAT and hyh deletion. [file Figure4.JPEG]
